# Bodily Information and Top-Down Affective Priming Jointly Affect the Processing of Fearful Faces

**DOI:** 10.3389/fpsyg.2021.625986

**Published:** 2021-06-02

**Authors:** Alessandra Nicoletta Cruz Yu, Pierpaolo Iodice, Giovanni Pezzulo, Laura Barca

**Affiliations:** ^1^Department of Psychological Science, Pomona College, Lincoln Hall, Claremont, CA, United States; ^2^Institute of Cognitive Neuroscience, Faculty of Brain Sciences, University College London, London, United Kingdom; ^3^Centre d’Etude des Transformations des Activités Physiques et Sportives (CETAPS), EA 3832, Faculty of Sports Sciences, University of Rouen, Mont Saint Aignan, France; ^4^Institute of Cognitive Sciences and Technologies, National Research Council, Rome, Italy

**Keywords:** emotional processing, interoception, affective priming, fear, mouse track analysis

## Abstract

According to embodied theories, the processing of emotions such as happiness or fear is grounded in emotion-specific perceptual, bodily, and physiological processes. Under these views, perceiving an emotional stimulus (e.g., a fearful face) re-enacts interoceptive and bodily states congruent with that emotion (e.g., increases heart rate); and in turn, interoceptive and bodily changes (e.g., increases of heart rate) influence the processing of congruent emotional content. A previous study by Pezzulo et al. (2018) provided evidence for this embodied congruence, reporting that experimentally increasing heart rate with physical exercise facilitated the processing of facial expressions congruent with that interoception (fear), but not those conveying incongruent states (disgust or neutrality). Here, we investigated whether the above (bottom-up) interoceptive manipulation and the (top-down) priming of affective content may jointly influence the processing of happy and fearful faces. The fact that happiness and fear are both associated with high heart rate but have different (positive and negative) valence permits testing the hypothesis that their processing might be facilitated by the same interoceptive manipulation (the increase of heart rate) but two opposite (positive and negative) affective primes. To test this hypothesis, we asked participants to perform a gender-categorization task of happy, fearful, and neutral faces, which were preceded by positive, negative, and neutral primes. Participants performed the same task in two sessions (after rest, with normal heart rate, or exercise, with faster heart rate) and we recorded their response times and mouse movements during the choices. We replicated the finding that when participants were in the exercise condition, they processed fearful faces faster than when they were in the rest condition. However, we did not find the same reduction in response time for happy (or neutral) faces. Furthermore, we found that when participants were in the exercise condition, they processed fearful faces faster in the presence of negative compared to positive or neutral primes; but we found no equivalent facilitation of positive (or neutral) primes during the processing of happy (or neutral) faces. While the asymmetries between the processing of fearful and happy faces require further investigation, our findings promisingly indicate that the processing of fearful faces is jointly influenced by both bottom-up interoceptive states and top-down affective primes that are congruent with the emotion.

## Introduction

Embodied theories of emotion suggest that emotional processing is at least in part grounded in bodily, interoceptive, and motor processes (Barsalou, 2008; Wilson-Mendenhall et al., 2013). Accordingly, every time an emotion is felt, thought about, or even recognized on someone else’s face, an *emotion simulation* would occur, which re-enacts perceptual, interoceptive, and motor streams congruent with (e.g., corresponding to past experiences of) the emotion in both the brain and the body (Oosterwijk et al., 2012). For example, the sight of fearful stimuli activates cortical areas involved in pain experience, including anterior cingulate cortex and insula (Botvinick et al., 2005) and amygdala (LeDoux, 2003; Garfinkel et al., 2014). In turn, the amygdala (via hypothalamus and brainstem circuits) engages autonomic processes and produces changes in heart rate (LeDoux, 2000).

Importantly, the re-enactment of interoceptive and bodily processes is not just a side-effect of emotional processing, but part and parcel of the emotional experience. Indeed, according to embodied theories, feelings and emotions may derive from a central representation of the physiological state of the body and its changes (James, 1884; Damasio and Carvalho, 2013). Embodied theories, therefore, predict that the momentary physiological condition of the body should influence the processing of emotionally charged stimuli. Early studies that experimentally induced states of high arousal and anxiety (e.g., by conducting the experiment on a fear-arousing bridge) reported that these physiological changes influenced subsequent ratings of attractiveness (Schachter and Singer, 1962; White et al., 1981). Additionally, the ingestion of different types of food has been found to influence the recognition of faces with different emotional expressions (Pandolfi et al., 2016). Other studies showed that manipulating facial expressions to render them congruent with specific emotions influenced the subsequent recognition of emotionally charged stimuli (McCanne and Anderson, 1987; Havas et al., 2010; Kever et al., 2015; Marmolejo-Ramos et al., 2020).

More recently, a study by Pezzulo and his colleagues manipulated (increased) heart rate before participants performed a gender-categorization task with (male and female) faces expressing an emotion congruent with the interoception of an increased heart rate (fear), an incongruent emotion (disgust), or neutrality (Pezzulo et al., 2018). Participants performed the task in two conditions: in a *rest condition*, wherein they remained at rest to maintain their baseline heart rate; and in an *exercise condition*, wherein they performed physical exercise to build and sustain an increased heart rate. Response time and kinematic variables were recorded using mouse-tracking software (Freeman et al., 2011). Pezzulo and his colleagues found that, despite the implicit nature of the gender-categorization task, an increased heart rate facilitated the participants’ processing of fearful faces, and not disgusted or neutral ones. These findings indicated that, when a certain kind of physiological condition (e.g., increased heart rate) is an interoceptive signature of a particular emotion simulation (e.g., fear), inducing the former facilitates the exteroceptive processing of stimuli displaying that emotion. This can be described as a form of *embodied congruence* between interoceptive state and emotional processing.

Such influence of the body’s physiological condition on emotional processing could be explained from the perspective of *interoceptive inference*, which posits that the brain uses interoceptive streams [e.g., signals from the internal organ as heart rate, breath, and gut (Craig, 2003)] to estimate the physiological condition of the body, to control corrective autonomic responses (Seth et al., 2012; Pezzulo, 2013; Seth, 2013; Barrett and Simmons, 2015; Pezzulo et al., 2015; Barrett et al., 2016; Seth and Friston, 2016; Gu et al., 2019). According to this framework, the resulting estimate of bodily and physiological parameters that culminates in the insula constitutes the central representation of the body’s state and its changes which, from an embodied perspective, is responsible for experiences of feeling and emotion (James, 1884; Damasio and Carvalho, 2013). Hence, an experimental manipulation such as physical exercise that increases heart rate (amongst with other interoceptive variables) would induce feelings and bodily responses that are congruent with the processing of fearful stimuli—therefore potentially facilitating the processing of these stimuli.

Pezzulo and his colleagues’ study, however, only addresses one aspect of interoceptive inference: the effect of (bottom-up) interoceptive signals such as heart rate in the processing of emotional states. Inferential theories of perception (Helmholtz, 1866; Gregory, 1980; Friston, 2005), including the theory of interoceptive inference, would predict *bottom-up* information (which, here, includes interoceptive streams such as heart rate) to be integrated with other *top-down* sources of information, such as the prior affective context (i.e., positive or negative), during the perception of emotionally charged stimuli. In other words, if emotional processing is the result of an inference, it should integrate multiple sources of bottom-up and top-down information and, thus, be influenced by both the physiological condition of the body—accessible via interoceptive streams—*as well as* the affective context in which the processing takes place. Whether and in which ways such integration of bottom-up and top-down information occurs during the processing of emotional stimuli has not been addressed thus far.

To address these questions, we designed the present study as an extension of Pezzulo and his colleagues’ experiment (Pezzulo et al., 2018), wherein we chose to manipulate not just, akin to the original study, participants’ (bottom-up) physiological conditions during a gender-categorization task, but also the (top-down) affective context by using *affective priming*.

Participants completed a gender-categorization task similar to Pezzulo and colleagues’ experiment (Pezzulo et al., 2018). Participants were presented with static pictures of faces showing positive (happy), neutral, and negative (fearful) facial expressions that they were asked to categorize as male or female. They performed the experiment in two conditions: in a *rest condition*, when they had remained at rest to maintain their baseline heart rate; and in an *exercise condition*, when they had performed physical exercise to achieve and sustain an increased heart rate. Similar to Pezzulo et al. (2018), mouse-tracking software was used to record response time and kinematic measures of the participants’ mouse trajectories while they completed the gender-categorization task. These recordings of the mouse movements provide more fine-grained information on the decision-making process with respect to the response time of button-pressing during the task. In particular, kinematic measures, such as the trajectory of the movement, provide information on the process of choice-revision while others, such as the x-flips or the smoothness of the trajectories across the horizontal axis, provide information on the participants’ uncertainty in making the choice and any “changes of mind” they might have had (Barca and Pezzulo, 2012, 2015; Flumini et al., 2015; Quétard et al., 2015, 2016; Barca et al., 2017; Iodice et al., 2017).

The main difference from the original study is that participants performed the task in three blocks (and twice, because they were tested both after rest and exercise). For each trial within the task, before receiving an image of a face with a fearful, neutral, or happy expression, they were shown an affective prime image, which was positive, neutral, or negative in these three different blocks. We chose affective priming as our prior context manipulation as previous studies have shown that manipulated emotional situations can be used to influence participants’ bottom-up processing of ambiguous stimuli (Dror et al., 2005). Moreover, affective stimuli, such as images or words, have been found to prime positivity and negativity in a subsequent task. An example of this phenomenon is when the valence of an affective prime matches the target of a task, bottom-up processing of that target was found to be quicker, even when the valence was irrelevant to the goal of the task, such as evaluating whether a string of letters was a real word or made-up (Fazio et al., 1986; Ferré and Sánchez-Casas, 2014).

Another difference with the original study is that we selected fearful, happy, and neutral faces, as opposed to fearful, disgusted, and neural ones. We did this to maintain symmetry with the three levels of our affective prime variable (i.e., fearful face and negative prime, happy face and positive prime, neutral face and neutral prime). Importantly, we selected positive (happiness) and negative (fearful) emotions which, despite their differing valences, are both associated to high heart rates—the physiological domain we are interested in. Indeed, despite inconsistent research on the interoceptive signatures of emotions, fear is widely associated with a number of physiological changes, including increased heart rate (Kreibig, 2010). Similarly, while the findings are not quite as unanimous as those on fear, there is a consensus that happiness is also associated with increased heart rate, especially when the stimuli are images (Dimberg and Thunberg, 2007).

This configuration of stimuli—i.e., emotional facial expressions with opposite affective valences (positive and negative), but shared physiological signature (increased heart rate)—allows us to test whether and how bottom-up and top-down processes are integrated. In line with previous findings (Pezzulo et al., 2018), we predicted the bottom-up manipulation (i.e., the increase of heart rate and other physiological signals via the exercise condition) to facilitate the processing of emotional (fearful and happy) faces compared to neutral faces – because fear and happiness are associated with similar bodily states (e.g., high heart rate) as the exercise condition. Furthermore, we predicted the top-down modulation (i.e., positive and negative primes) to further facilitate the processing of happy and fearful faces, respectively, when participants were in the exercise condition (and hence had high heart rate). This latter finding would imply that emotional processing could integrate bottom-up and top-down streams (here, changes of physiological state and emotional priming, respectively).

## Materials and Methods

### Ethics Statement

The study protocol conformed to the ethical guidelines of the Declaration of Helsinki (BMJ 1991; 302: 1194) as reflected in prior approval by the Institution of Cognitive Sciences and Technology’s human research committee (ISTC-CNR, Rome – N.0003971/04/12/2015).

### Participants

Thirty-six participants were recruited (*M*_*age*_ = 20 years old, *SD* = *0.90*; 50% females, 50% males) from University of Rouen Normandie, France. Participants were members of the same cultural group (i.e., Caucasian) and all were of French nationality. All participants were right-handed with normal or corrected-to-normal vision. All were volunteers and provided their informed consent before the experiment.

### Design and Stimuli

The study used a 2 (Physical Condition: rest, exercise) × 3 (Affective Priming: negative, neutral, positive) × 3 (Facial Expression: fearful, neutral, happy) within-subjects design.

*Stimuli of Affective Primes.* Participants were presented with positive, neutral, and negative images taken from the International Affective Picture System (IAPS) (Lang and Bradley, 2007). The images have been rated by valence, arousal, and dominance. Positive images have a valence range of 6.00–7.99, neutral images have a range of 4.00–5.99, and negative images of 2.00–3.99. Five hundred and forty images were used in total (i.e., 180 images for each valence level). Images from the IAPS were chosen because the picture system is standardized and its validity has been tested (Verschuere et al., 2001).

*Stimuli of Faces with Emotional Expressions.* Experimental stimuli comprised 45 male faces (15 happy, 15 neutral, 15 fearful emotional expressions) and 45 female faces (15 happy, 15 neutral, 15 fearful emotional expressions) from the Karolinska Directed Emotional Faces (KDEF) database (Lundqvist et al., 1998; Goeleven et al., 2008). The luminance of the images was normalized, and a Hann window was applied to remove hair and the peripheral information of the faces (Buck et al., 1972; Schwartz et al., 1980; Greenwald et al., 1989; Mermillod et al., 2009) and avoid gender-categorization based on the peripheral facial hair (Kaminski et al., 2011).

### Experimental Procedure

At the beginning of each trial, participants were instructed to click on the /START/ button located at the bottom-center of the screen. Then, a sequence of two stimuli were presented centrally: an affective prime (positive, neutral, or negative) for 200 ms, followed by a target face (male or female face, having either a happy, neutral or fearful expression) for 500 ms. Participants were instructed that their task was to perform a gender-categorization task: they had to categorize the target face as “Male” or “Female,” by clicking with the mouse on one of two response buttons, which appeared on the top-left or top-right corner of the screen. Rightward and leftward responses of “Male” and “Female” were counterbalanced. In each trial, the mouse could not be moved before the presentation of one of the target faces was completed.

While the participants were responding by moving their mouse, the x- and y- coordinates of the mouse trajectories were recorded (sampling rate of approximately 70 Hz) using the MouseTracker software (Freeman and Ambady, 2010). Given that each trial consists in a sequence of multiple stimuli (i.e., the prime for 200 ms and the face stimulus for 500 ms) we used a software command, which prevents mouse movements while the sequence of stimuli unfolds. Thus, the mouse “freezes” when the participant clicks on the /START/ button, and “unfreezes” after the last stimulus is presented.

Before the experimental block, participants received a block of 5 practice trials. Then, for the experimental task, they received 270 experimental trials in total, with 3 blocks of 90 trials each: one block with positive primes, one with neutral primes, and one with negative primes. Within each block, they received 90 primes and 90 face stimuli (30 happy faces, 30 neutral faces, and 30 negative faces, with 15 male and 15 female faces per emotion). They received the same set of face and prime stimuli for each block, in accordance with the within-subjects design. The blocks were counterbalanced among participants, and interleaved by a pause of one minute. Within each block, the trials were randomized.

All participants performed the gender-categorization task in two conditions on two different days at least one week apart from each other: an *exercise condition* session and a *rest condition* session. In the exercise condition, they were asked to bike on a cycle ergometer at their own rhythm for 3 min, both before the experimental task and during the pauses between blocks to ensure that the participants’ heart rates remained accelerated during the duration of the experimental task. In the rest condition, participants completed the experimental task without any prior physical activity. Instead of performing physical activity, they were asked to rest on their chair for 3 min before the task and for one minute during the pauses to maintain the timing across conditions. The order of rest and exercise conditions was counterbalanced across subjects.

A Polar RS800CX was used to check the participants’ heart rate after the rest or exercise period and before the task in each condition. When in the exercise condition, participants had an average of 122 ± 6 bpm, while when in the rest condition, they had an average of 69 ± 8 bpm.

### Mouse Tracker Data

Response time (RT) and accuracy were collected, together with parameters measuring mouse movement trajectories [i.e., trajectories Maximum Deviation (MD), and Area Under the Curve (AUC)].

Participants’ *response time* (RT) in milliseconds was recorded from the end of the presentation of the faces (i.e., when the mouse ‘‘unfroze’’)^1^ to the time they clicked on the selected gender option. Mouse *trajectory x-y coordinates* were automatically recorded by the software, indexing the location of the mouse along the horizontal and vertical axes.

The measure of *Maximum Deviation* (MD), calculated from the mouse coordinates, is the most substantial perpendicular deviation between the actual mouse trajectory and the ideal mouse trajectory (the straightest line from the start point to the endpoint of the trajectory), where positive scores indicate a trajectory in the direction of the unselected choice. Therefore, higher maximum deviation scores reflect participants’ maximal attraction to the unselected choice, while negative maximum deviation scores reflect the participant’s maximum attraction to the selected option.

The *Area Under the Curve* (AUC) is the space under the curve created by the actual trajectory in comparison to the ideal trajectory, where positive scores indicate a bend in the direction of the unselected choice. Therefore, higher scores reflect participants’ overall attraction to the unselected choice across all time-steps, while negative scores reflect the participant’s overall attraction to the selected option.

### Data Processing

Incorrect responses were removed from data analysis (0.03% of total data points). Response time outliers have been detected and removed with the R code “*outlierKD*,” which uses the Tukey’s method to identify the outliers ranged above and below the 1.5 ^∗^ interquartile range (Dhana, 2018).

Estimation statistics have been used to test and plot the effect size of the significant interactions. We computed and plotted Cohen’s *d* using the tools available at https://www.estimationstats.com/, determining the distribution with bootstrap sampling (5000 samples), and using bias-corrected and accelerated confidence intervals (Ho et al., 2019). An effect size of *d* = 0.2 is considered a “small” effect size, *d* = 0.5 represents a “medium” effect size, and *d* = 0.8 a “large” effect size. To ensure the robustness of our analyses, we (conservatively) only report large or medium effects.

## Results

### Response Time (RT)

**FIGURE 1:**
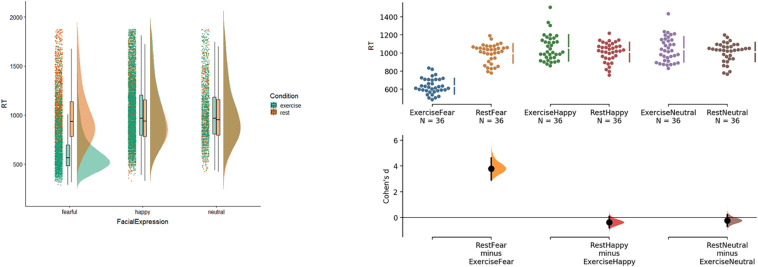
Response times for Condition and Facial Expression. Left: raincloud plots on correct response time. The plots visualize jittered raw data-points, box plots with median and extreme values, and probability distributions of correct response time for Condition and Facial Expression. Right: Cumming estimation plots and Cohen’s *d* on the same data. Raw data (response time in milliseconds) are plotted on the upper axes. Each mean difference is plotted on the lower axes as a bootstrap sampling distribution. Mean differences are depicted as dots; 95% confidence intervals are indicated by the ends of the vertical error bars. See the main text for explanation.

The overall response times, with the data from the three blocks aggregated, is displayed in Figure 1. The left part of Figure 1 shows raincloud plots [generated with the *cowplot* R script (Allen et al., 2018)], which highlight a difference in the distributions of response time for exercise and rest conditions for faces with a fearful expression. The right half of Figure 1, which depicts Cumming estimation plots and estimation statistics (i.e., Cohen’s *d*) on the same data, allows one to appreciate the result. The analysis shows that the exercise condition significantly speeds up the processing time for faces with fearful expressions (Cohen’s *d* = 3.78), but not happy (*d* = −0.3) or neutral ones (*d* = −0.2). Additionally, in the exercise condition, fearful faces were responded to significantly faster than faces with happy (*d* = 3.6) or neutral expressions (*d* = 3.5) (and *d*_*Happy–Neutral*_ = −0.12). In the rest condition, the differences are negligible (*d*_*Fear–Happy*_ = 0.14; *d*_*Fear–Neutral*_ = 0.15; *d*_*Happy–Neutral*_ = 0.01). No other comparisons appeared to be relevant. The facilitatory effect of the exercise condition on the processing of fearful but not neutral faces replicates the findings of Pezzulo et al. (2018). Contrary to our prediction, we did not find a facilitatory effect of the exercise condition on the processing of happy faces, despite happiness (similar to fear) being usually associated with increased heart rate.

**FIGURE 2:**
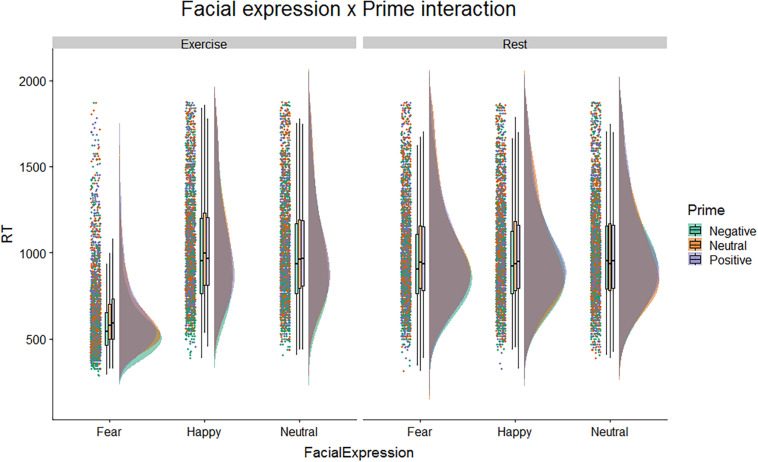
Raincloud plots on correct response time, for Physical Condition, Facial Expression, and Affective Prime.

After replicating the prior study’s results, we considered possible effects of affective primes on gender-categorization. Figure 2 shows raincloud plots of the response times in the different conditions. The figure highlights a difference in the distributions of response time for faces with a fearful expression with respect to faces with happy or neutral expression, but only in the exercise condition.

**FIGURE 3:**
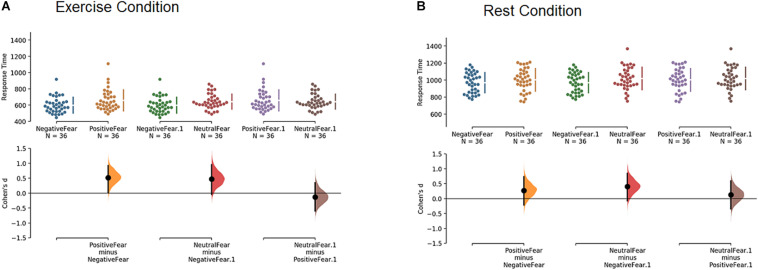
Cumming estimation plots and Cohen’s *d* on response time for the effect of the affective primes on fearful faces only, in exercise **(A)** and rest **(B)** conditions.

Figure 3 depicts this result in Cumming estimation plots and estimation statistics (i.e., Cohen’s *d*) on response time based on the effect of the affective primes, in exercise (panel A) and rest (panel B) conditions on fearful faces only (to avoid cluttering the figure).

In the exercise condition (panel A), participants responded faster to fearful faces when they were preceded by a negative prime compared to a positive (*d* = 0.52, large effect) or neutral (*d* = 0.47, small effect) prime. No difference emerged between positive and neutral primes (*d* = −0.13, trivial effect). In other words, when participants performed the experiment in the exercise condition, they responded to fearful faces significantly faster when the faces were preceded by a negative prime than a positive or neutral prime. The same comparisons in the rest condition (panel B) did not yield comparable results, with only a small effect (*d* = 0.40) for fearful faces preceded by negative vs. neutral primes. In keeping, a Mann-Whitney test indicates that the only two comparisons that reached statistical significance (P value < 0.05) were negative vs. positive primes and negative vs. neutral primes, but only when participants processed fearful faces in the exercise condition (the same comparisons failed to reach significance when participants were in the rest condition). For the sake of completeness, all the results of estimation statistics (i.e., Cohen’s *d*) and Mann-Whitney test are reported in Table 1. These results show that no other comparisons apart for those discussed above appeared to be relevant.

**TABLE 1:** Estimation statistics on Response times for for Condition, Facial Expression and Affective Prime.

	**Exercise**			**Rest**		
**Affective Prime**	**Fear**	**Happy**	**Neutral**	**Fear**	**Happy**	**Neutral**
Negative-Positive	0.52*	0.19	0.29	0.27	0.22	0.02
Negative-Neutral	0.47*	0.32	0.35	0.40	0.24	0.01
Positive-Neutral	–0.13	0.15	0.07	0.12	0.02	–0.01

Medium to large effects are bolded and underlined. Mann-Whitney test *P* value <0.05 are marked with *.

Finally, we conducted a further analysis to confirm that the effects of the negative prime are specific for fearful faces in the exercise condition. We found that in the presence of a negative prime, response times for feaful faces were faster in the exercise condition than in the rest condition (*d* = 3.41). However, no such effect was present for happy (d_*Exercise–Rest*_ = −0.26) or neutral faces (d_*Exercise–Rest*_ = 0.02).

### Movement Trajectories

**FIGURE 4:**
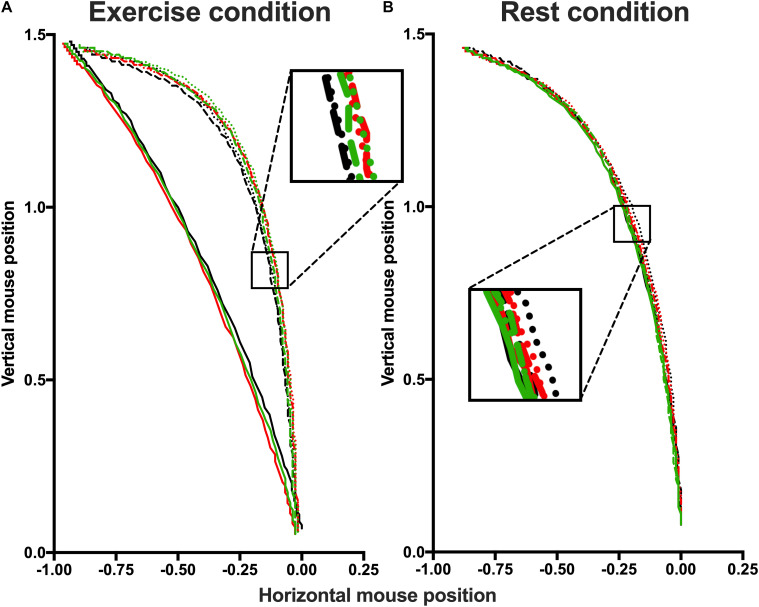
Spatial trajectories of the correct responses of exercise **(A)** and rest **(B)** conditions. In both conditions, trajectories are presented separately for each Affective prime and divided by Prime Facial Expressions: Negative Fearful (solid black line), Negative Happy (dashed black line), Negative Neutral (dotted black line); Neutral Fearful (solid red line), Neutral Happy (dashed red line), Neutral Neutral (dotted black line); Positive Fearful (solid green line), Positive Happy (dashed green line), Positive Neutral (dotted green line). The box highlight the difference between the curves. Participants in exercise conditions show a straighter trajectory for fearful faces but not for neutral and happy faces.

Mouse trajectories were rescaled into a standard coordinate space. The top-left corner of the screen corresponded to “−1, 1.5” and the bottom-right corner to “1, 0,” with the start location of the mouse (the bottom-center) with coordinates “0, 0.” Then, the duration of the trajectory movements were normalized by re-sampling the time vector into 101 time-steps using linear interpolation to allow averaging across multiple trials. Figure 4 displays average movement trajectories of correct responses for the different conditions, remapped leftward to enable comparisons. The figure shows that in the exercise (but not the rest) condition, mouse trajectories for fearful faces are significantly straighter compared to both neutral or happy faces—irrespective of the affective prime.

### Maximum Deviation (MD)

Estimation statistics on Maximum Deviation (MD) for Physical Condition and Facial Expression reveal that in the exercise condition MD values were lower in response to faces with fearful expressions (MD value = −0.75) compared to happy (MD value = 0.33, *d* = 4.96) or neutral ones (MD value = 0.37, *d* = 5.05), but were not different between happy and neutral faces (*d* = 0.29). On the contrary, in the rest condition, MD values were higher in response to faces with fearful expressions (MD value = −0.08) compared to happy (MD value = −0.001, *d* = 0.97) or neutral ones (MD value = 0.01, *d* = 1.23), but were not different between happy and neutral faces (*d* = 0.19). Note that the effect sizes were reduced in the rest compared to the exercise condition.

Table 2 reports the estimation statistics on Maximum Deviation (MD) for Physical Condition, Facial Expression and Affective Prime. The analysis indicates that, in the exercise condition, values of MD for happy faces were lower when the faces were preceded by a negative prime (MD value = 0.27) as opposed to a positive (MD value = 0.38) or neutral prime (MD value = 0.39), and values of MD for neutral faces were lower when preceded by a negative prime (MD value = 0.31) as opposed a positive one (MD value = 0.44). Other differences were negligible.

**TABLE 2:** Estimation statistics on trajectories’ Maximum Deviation.

	**Exercise**			**Rest**		
**Affective Prime**	**Fear**	**Happy**	**Neutral**	**Fear**	**Happy**	**Neutral**
Negative – Positive	0.09	**0.53**	**0.56**	–0.13	–0.09	–0.38
Negative-Neutral	0.08	**0.58**	0.39	0.09	0.16	–0.08
Positive – Neutral	–0.02	0.08	–0.18	0.25	0.27	0.35

*Medium to large effects are bolded and underlined.*

### Area Under the Curve (AUC)

Estimation statistics on Area Under the Curve (AUC) for Physical Condition and Facial Expression reveal that in the Exercise condition AUC values were smaller in response to faces with fearful expressions (AUC value = −0.52) compared to happy (AUC value = 0.21, *d* = 3.87) or neutral ones (AUC value = 0.25, *d* = 3.96), but were not different between happy and neutral faces (*d* = 0.29). On the contrary, in the rest condition, AUC values were larger in response to faces with fearful expresssions (AUC value = −0.08) compared to happy (AUC value = −0.0005, *d* = 0.93) or neutral ones (AUC value = −0.003, *d* = 0.92), but were not different between happy and neutral faces (*d* = −0.2). Note that the rest of the effect sizes were lower in the rest condition compared to the exercise one. Table 3 reports the estimation statistics on Maximum Deviation (MD) for Condition, Facial Expression and Affective Prime. The analysis reveals no medium or large size effects.

**TABLE 3:** Estimation statistics on Area Under the Curve.

	**Exercise**			**Rest**		
**Affective Prime**	**Fear**	**Happy**	**Neutral**	**Fear**	**Happy**	**Neutral**
Negative – Positive	0.09	0.418	0.36	–0.12	−0.24	–0.43
Negative – Neutral	0.04	0.48	0.42	–0.08	−0.05	–0.32
Positive – Neutral	–0.04	0.07	0.03	0.05	0.32	0.15

## Discussion

Embodied theories of emotion posit that the processing of emotionally charged stimuli consists of a situated simulation that re-enacts emotion-congruent physiological, interoceptive, and affective states (Wilson-Mendenhall et al., 2013; Barrett, 2017). Accordingly, not only should processing emotions with reliable somatic components, such as fear, produce significant physiological changes, such as heart rate acceleration (Cacioppo et al., 2000), but the converse should be true as well. For example, an accelerated heart rate should facilitate the processing of fearful faces—potentially via interoceptive channels that are key to the processing of bodily sensations and emotion (Kreibig, 2010; Barrett and Simmons, 2015).

This latter prediction was tested in a previous study by Pezzulo et al. (2018), which reported that after exercise (which increased heart rate), participants’ processing in gender-categorization of faces expressing fear was facilitated compared to that of faces expressing neutrality or disgust (Pezzulo et al., 2018). These results provided evidence for the bottom-up influence of both physiological and interoceptive states on emotional processing, specifically for affectively congruent pairs (increased heart rate and fear). That this effect was obtained in an incidental gender-categorization task is noteworthy, as it did not require participants to explicitly attend to the emotional content of the stimuli. This suggests that emotional content is nevertheless implicitly attended to.

In the present study, we aimed to replicate this finding and extend it to study the joint-effect of the above (bottom-up) interoceptive manipulation of heart rate and a (top-down) manipulation of the context of emotional processing via affective priming. To this end, we tested participants after rest and exercise in the gender-categorization of faces with three types of emotional expressions: two (happiness and fear) both congruent with high heart rate, but with opposite valence (positive and negative) and the third, neutral. Participants performed the gender-categorization in three blocks (for each affective priming condition), during which emotional faces were preceded by positive or negative primes (congruent with happy and fearful faces, respectively) or neutral primes.

Theories of *interoceptive or embodied inference* would interpret such emotional processing as the result of a principled (Bayesian) inferential process that integrates (top-down) affective primes and (bottom-up) interoceptive streams (Seth et al., 2012; Pezzulo, 2013; Seth, 2013; Barrett and Simmons, 2015; Pezzulo et al., 2015; Barrett et al., 2016; Seth and Friston, 2016; Gu et al., 2019). From these perspectives, positive and negative primes should have facilitated the processing of happy and fearful faces, respectively, when participants were in an emotion-congruent bodily state (i.e., increased heart rate). This would be explained by both the interoceptive information of increased heart rate and the priming of positive (or negative) emotional content anticipating the incoming perception of happy (or fearful) faces. This anticipation would facilitate the processing of those interoceptively and prior affectively congruent stimuli. In more ecological contexts, interoception and prior affective context could be used to to prepare to deal with behaviorally relevant positive or negative events (Seth et al., 2012; Pezzulo, 2013; Seth, 2013; Barrett and Simmons, 2015; Pezzulo et al., 2015; Barrett et al., 2016; Seth and Friston, 2016; Friston et al., 2017; Gu et al., 2019).

Our results replicated the *embodied congruence* between an interoceptive state of high heart rate^2^ and emotional target reported in Pezzulo et al. (2018). Participants in the exercise condition processed fearful faces *faster* than the other faces (see Figure 1). While the response time values reported in the present study seem shorter than those in the original one, the response times between these two studies are comparable as, in this study, the mouse was frozen until the end of the presentation of the face, while this was not the case in the previous one. Furthermore, akin to Pezzulo et al. (2018), trajectories toward fearful faces in the exercise condition had *lower* MD and AUC values compared to those in response to other facial expressions (see Figure 3). Surprisingly, trajectories toward fearful faces had *higher* MD and AUC values in the rest condition compared to the other faces. While this difference was not significant in Pezzulo et al., it trended in that direction (Pezzulo et al., 2018).

Our results extend the findings of Pezzulo et al. (2018) by showing that, in accordance with our hypothesis, participants in the exercise condition processed fearful faces even faster when they were preceded by a negative prime compared to the other primes (see Figure 2). However, contrary to our hypothesis, participants did not process happy faces faster when they were in the exercise compared to the rest condition. Additionally, participants in the exercise condition did not process happy faces faster when they were preceded by positive primes. Thus, our findings suggest that emotional processing integrates (top-down) affective context and (bottom-up) interoceptive condition of the body, but only in the processing of fearful faces.

These asymmetric results—and the fact that neither exercise nor positive primes alone, or their combination, facilitated the processing of happy faces—may be due to a number of factors. First, both fearful and happy faces are associated to high heart rate, but the latter less consistently, which may explain why the high heart rate induced by physical exercise facilitates the processing of fearful, but not happy faces. A further (not mutually exclusive) possibility is that positive IAPS primes (Lang and Bradley, 2007) exerted a weaker effect compared to negative primes on the processing of emotional stimuli. The prominence of negative primes is supported by the analysis of kinematic measures (maximum deviation, MD), which shows that in the increased heart rate condition, negative primes influenced the processing of happy and, to some extent, neutral faces, reducing the attractiveness of competing alternatives. The lack of an effect on fearful faces could plausibly be due to a ceiling effect, given that the MD of trajectories with fearful faces is already extremely low. Future studies using a wider range of emotional expressions and primes may help establish to what extent the asymmetry of results between the positive and negative domains—and the stronger effect of negative vs. positive primes—depend on the materials we used or fundamental differences between emotional domains.

In summary, the analyses of response times and movement kinematics suggest that the congruence between negative primes and increased heart rate influences the emotional processing in two ways. The first is valence-specific: as reported above, it selectively *speeds up* the processing of fearful stimuli, which are congruent with both high heart rate and negative primes. This *embodied congruence* between interoceptive state, affective priming, and emotional expression processing is compatible with Pezzulo et al. (2018) as well as theories of *interoceptive or embodied inference* which predict that (top-down) negative affective primes and (bottom-up) interoceptive streams signaling high heart rate would selectively influence the processing of fearful faces (Seth et al., 2012; Pezzulo, 2013; Seth, 2013; Barrett and Simmons, 2015; Pezzulo et al., 2015; Barrett et al., 2016; Seth and Friston, 2016; Gu et al., 2019). The second may consist of a broader form of arousal, which non-selectively influences the processing of happy and neutral faces, too. Previous research has reported that arousal from exercise can facilitate emotional processing, improving vigilance and performance in cognitive tasks (Poulton, 1977). Our results suggest that valence-specific and broader effects are not mutually exclusive, but can coexist. However, the latter, broader effects seem to be more limited and confined to some subtle kinematic aspects of movement.

There are some limitations in the present study that can be corrected or expanded on in further studies. First, as noted above, it is possible that our materials of happy and fearful faces, or positive and negative primes, were not completely balanced. This is something that future studies should carefully control. Second, we used a block design and this may have limited the effect of affective priming. Future experiments using an event-related rather than a block design may be more effective in testing significant effects of the prime on emotion processing. Third, the limited sample of participants prevented us from analyzing the gender variable across participants. In addition, we did not design the study to test gender differences and we do not have *a priori* hypotheses about this comparison (especially in this task, which is a gender classification task that probes emotional processing indirectly). Fourth, a wider variety of emotions should be tested. Further categories of facial expression should be used to expand our repertoire of potential interoceptive signatures of different emotions. Different emotional target stimuli could also be used to investigate more granular types of emotion, such as nostalgia, annoyance, or sexual arousal. Such target stimuli could consist of images, videos, or stories of specifically emotionally salient situations. Fifth, a greater variety of ethnicities should also be introduced in further experimentation by having participants consist of different ethnic groups and the face stimuli as well. Finally, future studies may consider applying this paradigm to clinical populations, given the emerging link between interoceptive dysfunctions and several psychopathological conditions such as anorexia nervosa (Barca and Pezzulo, 2020), bulimia nervosa (Klabunde et al., 2017), anxiety and addiction (Paulus et al., 2019), somatic symptoms perception (Pezzulo et al., 2019), and panic disorders (Maisto et al., 2021).

Despite these limitations, the present study provides promising evidence that the implicit processing of fearful facial expressions is facilitated by both congruent interoceptive states and affective primes within the same paradigm. It shows that salient interoceptive signals, such as a high heart rate and negative affective prime, jointly create the appropriate context—or expectation—to process subsequent congruent stimuli such as a fearful face.

## Data Availability Statement

The raw data supporting the conclusions of this article will be made available by the authors, without undue reservation.

## Ethics Statement

The studies involving human participants were reviewed and approved by Institution of Cognitive Sciences and Technology’s Human Research Committee (ISTC-CNR, Rome – N.0003971/04/12/2015). The patients/participants provided their written informed consent to participate in this study.

## Author Contributions

ANCY, PI, GP, and LB designed the research. PI performed the research. LB analyzed the data. GP and LB wrote the article in consultation with ANCY and PI. All authors contributed to the article and approved the submitted version.

## Conflict of Interest

The authors declare that the research was conducted in the absence of any commercial or financial relationships that could be construed as a potential conflict of interest.

## Footnotes

1 Given that we used “compound trials” of two stimuli (a prime for 200 ms and a face stimulus for 500 ms) sequentially presented, a software command was used to block mouse movement during the unfolding of the stimulus sequence. Therefore, the mouse was frozen from the moment the participant pressed the /START/ button and only unfroze once the face stimulus presentation was completed. This procedure prevented the recording of accidental micro-movements of the mouse that may occur before the face stimulus appears or can be processed.

2 In our discussion of embodied congruence, we focus on the fact that our manipulation (physical exercise) produces significantly higher heart rates compared to the rest condition; and specifically interoceptive signals of high heart rate may explain the facilitation of fearful face processing. However, physical exercise has several other interoceptive effects in addition to increased heart rate, such as for example increased body temperature, fast breathing, or even differences in pH in the gut. In principle, any combination of these interoceptive signals may produce an embodied congruence with fearful faces. Nevertheless, in the following interpretations, we continue to refer to heart rate, as this is an index that we explicitly measured during the experiment.
